# The complete chloroplast genome of *Beesia deltophylla* (Ranunculaceae)

**DOI:** 10.1080/23802359.2021.1972873

**Published:** 2021-09-17

**Authors:** Zhe-Fei Zeng, Rui Rao, Qing-Guo Yu, Liang-Liang Yue

**Affiliations:** Yunnan Key Laboratory of Plateau Wetland Conservation, Restoration and Ecological Services, Southwest Forestry University, Kunming, China

**Keywords:** *Beesia deltophylla*, chloroplast genome, phylogenetic analysis

## Abstract

*Beesia deltophylla* is an endemic and rare species only distributed in Xizang, China. The chloroplast genome of *B. deltophylla* is 157,397 bp in length, with 112 encoded genes including 78 protein-coding genes, 30 tRNA genes and 4 rRNA genes. Phylogenetic reconstruction has confirmed the placement of *B. deltophylla* as sister to *B. calthifolia*. These two species formed a clade closely to a Japan endemic species *Anemonopsis macrophylla*.

*Beesia deltophylla* C. Y. Wu is a buttercup species endemic in Xizang, China (Xiao 1979). It has a sister species, *B.calthifolia,* mainly distributed in Southwest China. Previous studies have confirmed this genus as a monophyletic group related to the *Anemonopsis* in Cimicifugeae (Yang et al. 1995, Wang et al. 1998, Gao et al. 2008). The genus *Beesia* is also a traditional Chinese medicine resource. The population size of *B. deltophylla* is extremely small and was situated in an extremely small population in the Red List of endangered species (Vié et al. 2009). At present, the chloroplast genome of *B. calthifolia* has been published (Zhai et al. 2019). Here, we report the complete chloroplast genome of *B. deltophylla* and reconstruct phylogenetic relationship to provide more molecular sequences for further research.

The samples of *B. deltophylla* were collected from Xizang China (29°22′ N, 95°8′ E). A specimen was deposited at Wetland College of Southwest Forestry University (http://plateauwetland.swfu.edu.cn/, Liang-Liang Yue, email: yueliangliang@swfu.edu.cn) under the voucher number yue20190135. A sequencing library was sequenced using Illumina nova-seq 6000 platform. NGS QC ToolKit (Patel and Jain 2012) was utilized to filter all raw readings and obtain high-quality clean reads. The complete chloroplast genome was assembled using NOVOPlasty (Dierckxsens et al. 2017). The chloroplast genome of *B. calthifolia* (GenBank accession number: NC_041531) was used as a reference sequence for assembling. After annotating the genome using GeSeq (Tillich et al. 2017), GB2squine (Lehwark and Greiner 2019) was utilized to convert the generated chloroplast genome annotation files into feature table files and submitted to GenBank via online BankIt.

The chloroplast genome of *B. deltophylla* has a full length of 157,397 bp. It is generally presented as a typical quartile structure, consisting of a large single-copy region (LSC, 86,863 bp), a small single-copy region (SSC, 17,481 bp), and a pair of inverted repeat regions (IRa/b, 26,525 bp). The chloroplast genome contains 112 genes, including 78 protein-coding genes, 30 tRNA genes, and 4 rRNA genes. The GC content of the *B. deltophylla* chloroplast genome was 38% and the corresponding values in LSC, SSC, and IR regions were 36.6%, 32.6%, 43.2%, respectively. Annotated chloroplast genome sequence was submitted to GenBank with an accession number MZ350960.

The phylogenetic analysis was based on *B. deltophylla* and other 22 complete chloroplast genomes published on NCBI, using the *Dysosma versipellis* as the outgroup. All sequences were aligned by MAFFT v7 (Katoh and Standley 2013) and manually adjusted in MEGA 7.0 (Kumar et al. 2016). The most suitable nucleotide substitution model is determined by ModelFinder (Kalyaanamoorthy et al. 2017), and the best model is determined as GTR + F+R4. Then use IQ-TREE 1.62 (Nguyen et al. 2015) to perform maximum likelihood (ML) analysis based on the best model and 5000 ultra-fast bootloaders . The ML tree showed that *Beesia* is a monophyletic group with a 100% bootstrap value (Figure 1). Our results provide basic information for further phylogenetic and biogeographic researches on the *Beesia* and the *Cimicifugeae*.

**Figure 1.**
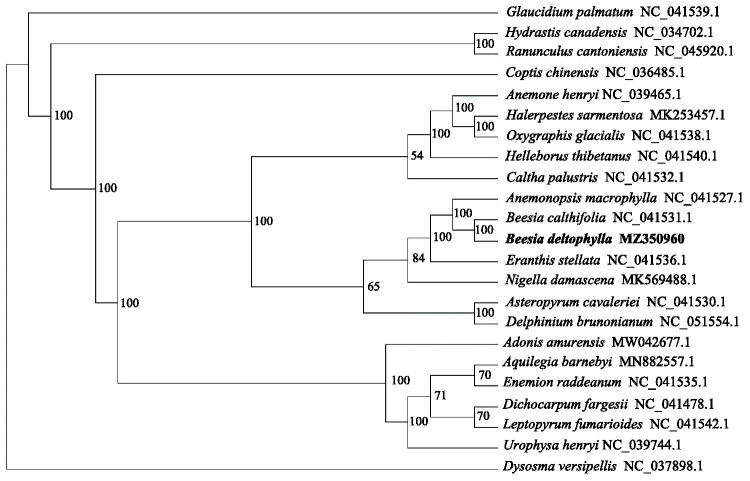
Maximum likelihood (ML) phylogenetic tree based on 23 complete chloroplast genomes. The tree was rooted using *Dysosma versipellis*, Berberidaceae, as outgroup. The bootstrap support values were marked above the branches.

## Data Availability

The genomic sequence data supporting the results of this study can be obtained in GenBank of NCBI at (https://www.ncbi.nlm.nih.gov) under the accession number MZ350960. The associated BioProject, SRA, and Bio-Sample numbers are PRJNA744067, SRR15048188, and SAMN20079128, respectively.
